# PHD2 inactivation in Type I cells drives HIF‐2α‐dependent multilineage hyperplasia and the formation of paraganglioma‐like carotid bodies

**DOI:** 10.1113/JP275996

**Published:** 2018-08-19

**Authors:** James W. Fielding, Emma J. Hodson, Xiaotong Cheng, David J. P. Ferguson, Luise Eckardt, Julie Adam, Philomena Lip, Matthew Maton‐Howarth, Indrika Ratnayaka, Christopher W. Pugh, Keith J. Buckler, Peter J. Ratcliffe, Tammie Bishop

**Affiliations:** ^1^ Target Discovery Institute University of Oxford, Oxford, UK; ^2^ Ludwig Institute for Cancer Research University of Oxford, Oxford, UK; ^3^ John Radcliffe Hospital University of Oxford, Oxford, UK; ^4^ Department of Physiology, Anatomy and Genetics University of Oxford Oxford UK; ^5^ The Francis Crick Institute London UK

**Keywords:** hypoxia, HIF, HIF prolyl hydroxylase (PHD)

## Abstract

**Key points:**

- The carotid body is a peripheral arterial chemoreceptor that regulates ventilation in response to both acute and sustained hypoxia.
- Type I cells in this organ respond to low oxygen both acutely by depolarization and dense core vesicle secretion and, over the longer term, via cellular proliferation and enhanced ventilatory responses.
- Using lineage analysis, the present study shows that the Type I cell lineage itself proliferates and expands in response to sustained hypoxia.
- Inactivation of HIF‐2α in Type I cells impairs the ventilatory, proliferative and cell intrinsic (dense core vesicle) responses to hypoxia.
- Inactivation of PHD2 in Type I cells induces multilineage hyperplasia and ultrastructural changes in dense core vesicles to form paraganglioma‐like carotid bodies.
- These changes, similar to those observed in hypoxia, are dependent on HIF‐2α.
- Taken together, these findings demonstrate a key role for the PHD2–HIF‐2α couple in Type I cells with respect to the oxygen sensing functions of the carotid body.

**Abstract:**

The carotid body is a peripheral chemoreceptor that plays a central role in mammalian oxygen homeostasis. In response to sustained hypoxia, it manifests a rapid cellular proliferation and an associated increase in responsiveness to hypoxia. Understanding the cellular and molecular mechanisms underlying these processes is of interest both to specialized chemoreceptive functions of that organ and, potentially, to the general physiology and pathophysiology of cellular hypoxia. We have combined cell lineage tracing technology and conditionally inactivated alleles in recombinant mice to examine the role of components of the HIF hydroxylase pathway in specific cell types within the carotid body. We show that exposure to sustained hypoxia (10% oxygen) drives rapid expansion of the Type I, tyrosine hydroxylase expressing cell lineage, with little transdifferentiation to (or from) that lineage. Inactivation of a specific HIF isoform, HIF‐2α, in the Type I cells was associated with a greatly reduced proliferation of Type I cells and hypoxic ventilatory responses, with ultrastructural evidence of an abnormality in the action of hypoxia on dense core secretory vesicles. We also show that inactivation of the principal HIF prolyl hydroxylase PHD2 within the Type I cell lineage is sufficient to cause multilineage expansion of the carotid body, with characteristics resembling paragangliomas. These morphological changes were dependent on the integrity of HIF‐2α. These findings implicate specific components of the HIF hydroxylase pathway (PHD2 and HIF‐2α) within Type I cells of the carotid body with respect to the oxygen sensing and adaptive functions of that organ.

## Key points

- The carotid body is a peripheral arterial chemoreceptor that regulates ventilation in response to both acute and sustained hypoxia.
- Type I cells in this organ respond to low oxygen both acutely by depolarization and dense core vesicle secretion and, over the longer term, via cellular proliferation and enhanced ventilatory responses.
- Using lineage analysis, the present study shows that the Type I cell lineage itself proliferates and expands in response to sustained hypoxia.
- Inactivation of HIF‐2α in Type I cells impairs the ventilatory, proliferative and cell intrinsic (dense core vesicle) responses to hypoxia.
- Inactivation of PHD2 in Type I cells induces multilineage hyperplasia and ultrastructural changes in dense core vesicles to form paraganglioma‐like carotid bodies.
- These changes, similar to those observed in hypoxia, are dependent on HIF‐2α.
- Taken together, these findings demonstrate a key role for the PHD2–HIF‐2α couple in Type I cells with respect to the oxygen sensing functions of the carotid body.

## Introduction

Acclimatization is an important physiological response to low oxygen levels and the ventilatory component of this response is mediated by the carotid body (CB), a peripheral chemoreceptor (Bisgard, 2000; Robbins, 2007; Kumar & Prabhakar, 2012; Lopez‐Barneo *et al*. 2016). Previous work has implicated the HIF system in the modulation of CB function (Kline *et al*. 2002; Peng *et al*. 2011; Bishop *et al*. 2013). HIF is regulated by a series of 2‐oxoglutarate‐dependent dioxygenases that generate an oxygen‐dependent signal by the catalysis of hydroxylation on specific prolyl residues in HIFα subunits (Bishop & Ratcliffe, 2014). Mammalian cells express three closely related HIF prolyl hydroxylases: PHD1, 2 and 3, of which PHD2 is the most abundant and important regulator in most cells. Similarly, HIFα is represented in mammalian species by three isoforms: HIF‐1α, HIF‐2α and HIF‐3α. HIF‐1α and HIF‐2α are the most abundant and the best studied. Despite many similarities in their structure and regulation by the PHDs, increasing evidence indicates that they have distinct transcriptional targets and physiological roles (Schodel *et al*. 2011; Ratcliffe *et al*. 2017). In recent work, we have observed that general inactivation of HIF‐2α, but not HIF‐1α, ablates the proliferation that occurs within days of exposure to hypoxia in adult mice (Hodson *et al*. 2016).

Whilst this and other work has implicated the HIF hydroxylase ‘oxygen sensing’ pathway in this response, it leaves open the question of which cell or cells are responsible for the effects, and whether and under what circumstances activation of HIF in one or more specific cell types is sufficient to drive proliferation and expansion of the CB. These questions are important both for understanding the physiological mechanisms by which hypoxia signalling pathways impinge on CB physiology, as well as for understanding the pathophysiology of CB paraganglioma, a tumour that is more commonly observed (10‐fold) in individuals living at altitude (Saldana *et al*. 1973). Because hypoxia pathways are commonly activated in cancer (Ratcliffe, 2013; Kaelin, 2017), these questions may also be of general relevance to that disease.

Although labelling of DNA synthesis with bromodeoxyuridine (BrdU) in cells that were identified as Type I (based on morphological appearances and the expression of an immunohistochemical marker) has been interpreted as evidence for the cell‐autonomous proliferation of that population (Paciga *et al*. 1999; Nurse & Fearon, 2002; Wang & Bisgard, 2002; Wang *et al*. 2008), other studies have suggested that such cells might arise mainly by proliferation within a stem cell niche of glia‐like sustentacular cells followed by subsequent transdifferentiation (Pardal *et al*. 2007).

To study this process further, we have applied lineage tracing technology, together with cell‐specific inactivation of particular components of the HIF hydroxylase pathway. We report that exposure to 28 days of hypoxia in the mouse promotes the proliferation and expansion of lineage labelled Type I cells of the CB. HIF‐2α inactivation in the Type I cells of the CB severely reduced both hypoxia‐induced CB proliferation and ventilatory acclimatization to hypoxia. Furthermore, inactivation of the principal HIF prolyl hydroxylase PHD2 within Type I cells was sufficient to drive the growth of greatly enlarged and highly vascular CBs via mechanisms that are dependent on HIF‐2α.

Our findings provide insights into the action of hypoxia on growth of the CB and suggest a model for the study of hypoxia‐associated paraganglioma.

## Methods

### Ethical approval and animals

Animal procedures were approved by the University of Oxford Medical Sciences Division Ethical Review Committee and were compliant with the UK Home Office Animals (Scientific Procedures) Act 1986. The authors understand the ethical principles of *The Journal of Physiology* and all work was conducted in compliance with stated standards (Grundy, 2015). Mice were housed in individually ventilated cages and fed *ad libitum* on a standard diet. Male mice aged ∼3 months old were used for all comparisons with littermate matched controls, except where stated otherwise. *Phd2^f/f^*, *Hif‐1α^f/f^* and *Hif‐2α^f/f^* (where ‘f’ denotes the floxed allele) conditional knockout have been described previously and were obtained from these sources (Cramer *et al*. 2003; Gruber *et al*. 2007; Mazzone *et al*. 2009). Mouse lines in which tamoxifen regulated Cre recombinase is expressed from the tyrosine hydroxylase (TH) (*TH‐IRES‐CreER*; hereafter referred to as *THCreER*) and glial fibrillary acidic protein (GFAP) (*GFAPCreERT2*; hereafter referred to as *GFAPCreER*) promoters have been described previously (Hirrlinger *et al*. 2006; Rotolo *et al*. 2008). The Ai14 *tdTomato* reporter line has been described previously, demonstrating robust fluorescence following activation by Cre recombinase (Madisen *et al*. 2010). Each line had been backcrossed with C57BL/6 for at least five generations and was intercrossed to generate littermates of appropriate genotypes. The mouse line with Cre recombinase expressed from the TH promoter via an internal ribosomal entry sequence (*TH‐IRES‐Cre*; hereafter referred to as *THCre*) has been described previously, demonstrating expression in catecholaminergic neurones. In this line, some recombination has been described in germ cells, leading to constitutive recombination in offspring (Lindeberg *et al*. 2004). Therefore, all mice used for mating and experiments were screened for germline recombination using ear biopsy samples and affected animals were eliminated from the study. PCR primers used to test for recombination are as described in Adam *et al*. (2011).

### Chronic hypoxic exposure of mice and drug administration

Tamoxifen (prepared to 20 mg mL^−1^ in corn oil containing 10% ethanol; Sigma, St Louis, MO, USA) was administered by oral gavage to ∼6 week‐old mice at a dose of 2 mg per day for 5 consecutive days as described previously (Arsenault *et al*. 2013; Hodson *et al*. 2016). After a 5 day tamoxifen ‘washout’ period (*t* = 0 days), littermate mice were split into hypoxia and normoxia control groups. Mice were administered 50 mg kg^−1^ BrdU via i.p. injection immediately prior to exposure to hypoxia or normoxia, followed by supplementation of drinking water with BrdU (1 mg mL^−1^ BrdU and 1% sucrose) for the duration of the experiment. Hypoxic mice were placed into a normobaric altitude chamber and acclimatized to decreasing oxygen levels over 24 h, and then held at 10% oxygen for 7 or 28 days with controlled temperature, humidity and carbon dioxide levels and free access to food and water. Normoxic littermate controls were maintained under normal housing conditions and supplemented with BrdU water (1 mg mL^−1^ BrdU and 1% sucrose). All mice were weighed at the start of the procedure and monitored daily for general condition and changes in body weight. No significant differences in body weight or surface body temperature (assessed by thermal imaging using an infrared camera, PI 160; Optris, Berlin, Germany) were noted in *Hif‐2α^f/f^*;*THCreER* mice *vs*. *Hif‐2α^f/f^* littermate controls during the hypoxic exposure.

### Plethysmography

Tidal volume and respiratory rate were measured in awake, unrestrained mice using individual whole body plethysmographs (600 mL volume; Model PLY4211; Buxco, DSI, St Paul, MI, USA) (Bishop *et al*. 2013; Hodson *et al*. 2016). Minute ventilation was calculated from tidal volume and respiratory rate. Ventilatory parameters were derived using FinePointe software (Buxco) and adjusted to body weight as measured immediately prior to plethysmography. Premixed gas was delivered to each chamber as described previously (Bishop *et al*. 2013; Hodson *et al*. 2016). The acute hypoxic stimuli consisted of 10% oxygen, balance nitrogen or 10% oxygen, 3% carbon dioxide, balance nitrogen. The hypoxic ventilatory response (HVR) to each stimulus was defined as the difference between minute ventilation during the 1 min prior to the onset of hypoxia and the first 1 min of stable hypoxia (Bishop *et al*. 2013; Hodson *et al*. 2016). Ventilatory instabilities (such as apnoeas and periodic breathing) were noted in the immediate post‐hypoxic period, as reported previously for mice on a C57BL/6 genetic background (Han *et al*. 2001, 2002). However, no differences in the frequency of these events were noted between the genotypes tested.

### Blood measurements

Blood was taken from the inferior vena cava of mice using heparinized capillary tubes after terminal anaesthesia, and haematocrits were measured using a haematocrit centrifuge (model C‐MH30; Unico, Dayton, NJ, USA).

### Tissue preparation

Mice were killed by overdose of isoflurane and exsanguination when still in their experimental oxygen condition (i.e. hypoxic mice were culled in the altitude chamber). This was followed by immediate perfusion fixation and dissection of tissues that were then immersed in 4% paraformaldehyde/PBS overnight. Tissues were then transferred to 30% sucrose/PBS or 70% ethanol prior to processing and embedding. Tissues for immunofluorescence were embedded in OCT embedding compound and cryosectioned to an 8 μm thickness using a CM‐3050‐S Cryostat (Leica Biosystems, Wetzlar, Germany). Paraffin‐embedded tissues were sectioned to a 4 μm thickness using an RM2135 microtome (Leica Biosystems).

### Immunohistochemistry and *in situ* hybridization

Cryosections were immunostained with rabbit anti‐TH antibody (dilution 1:500; NB300‐109; Novus Biologicals, Cambridge, UK), rabbit anti‐GFAP (dilution 1:500; ZO334; Dako, Ely, UK), rat anti‐endomucin (dilution 1:500; sc‐65495; Santa Cruz Biotechnology, Santa Cruz, CA, USA) and sheep anti‐BrdU (dilution 1:500; ab1893; Abcam, Cambridge, UK), followed by detection with an Alexafluor 488 goat anti‐rabbit (dilution 1:500; #R37116; Thermo Fisher Scientific, Loughborough, UK) or an Alexafluor 488 goat anti‐rat (1:500; A11006; Thermo Fisher Scientific) secondary antibody and a 4′,6‐diamidino‐2‐phenylindole nuclear counterstain (dilution 1:2000; ab104139; Abcam). Paraffin‐embedded tissues were immunostained with anti‐TH (dilution 1:5000; NB300‐109; Novus Biologicals) or with an anti‐BrdU antibody in accordance with the manufacturer's instructions (dilution 1:10; #551321; Becton Dickinson Biosciences, Oxford, UK) as described previously (Bishop *et al*. 2013; Hodson *et al*. 2016).

*Hif‐1α*, *Hif‐2α* and *Vegfa* transcripts were detected via *in situ* hybridization on sections from paraffin‐embedded tissues using the RNAscope manual assay in accordance with the manufacturer's instructions (Advanced Cell Diagnostics, Newark, CA, USA). Loss of *Hif‐2α* expression in mice with conditional gene inactivation was demonstrated using the BaseScope kit (Advanced Cell Diagnostics) and a customized probe targeted to exon 2 (Advanced Cell Diagnostics), which is flanked in the *Hif‐2α* floxed allele by LoxP sites and hence absent in any transcripts from cells where Cre mediated recombination has taken place (Gruber *et al*. 2007). To obtain dual *in situ* hybridization/immunohistochemically stained images, the RNAscope protocol was performed first followed by immunostaining with anti‐TH or anti‐BrdU antibodies as described above. The percentage of TH^+^ cells expressing *Hif‐2α* mRNA was quantified to give a measure of the efficiency of tamoxifen‐induced recombination in Type I cells throughout the CB.

Samples were imaged using a model 710 confocal microscope or a DM 1000 LED microscope (Leica Biosystems). Stereological estimation of cell number, cell density and CB volume was performed using ImageJ (NIH, Bethesda, MD, USA) on alternate cryosections or on every fourth paraffin section as described previously (Bishop *et al*. 2008; Bishop *et al*. 2013; Hodson *et al*. 2016).

### Electron microscopy

The bifurcation of the carotid artery, containing the CB, was removed and immediately placed in 4% glutaraldehyde in 0.1 m phosphate buffer and processed for routine electron microscopy. The tissue was post‐fixed in osmium tetroxide, treated *en bloc* with uranyl acetate, dehydrated in ethanol and then treated with propylene oxide and embedding epoxy resin. Sections (1 μm) were cut from the blocks and examined by light microscopy to identify the location of the CB within the bifurcation. Thin sections of selected areas were stained with uranyl acetate and lead citrate prior to examination with a transmission electron microscope (JEOL 1200EX; JEOL, Tokyo, Japan). The approximate area of Type I cells was calculated from electron micrographs of centrally sectioned cells using ImageJ.

### Statistical analysis

Data are shown as the mean ± SEM. Statistical analyses were performed using unpaired Student's *t* tests. For repeated measures, data were analysed by ANOVA followed by Tukey's multiple comparison test or *t* test with Holm–Sidak correction for multiple comparisons as appropriate and as described in Hodson *et al*. (2016). *P* < 0.05 was considered statistically significant.

## Results

### The effect of hypoxia on Type I and II lineage labelled cell populations in the CB

To assess which populations of cells in the CB proliferate in response to hypoxia, specific cell types were labelled in normoxic animals using an inactive Ai14 *tdTomato* reporter gene (Madisen *et al*. 2010). This gene was recombined into the ubiquitously expressed Rosa26 locus and then activated in specific lineages by cell type specific expression of Cre recombinase, allowing the marking of cells and tracing of their progeny by tdTomato fluorescence. Inducible Cre recombinase transgenes were selected to permit timed cell‐specific expression following exposure to tamoxifen: a TH promoter transgene (*THCreER*) whose expression is restricted within the CB to Type I (glomus) cells (Rotolo *et al*. 2008; Macias *et al*. 2014) and a GFAP promoted transgene (*GFAPCreER*) whose expression is restricted to glia‐like support cells (Hirrlinger *et al*. 2006), such as the Type II cells of the CB (Kameda, 2005).

**Figure 1:**
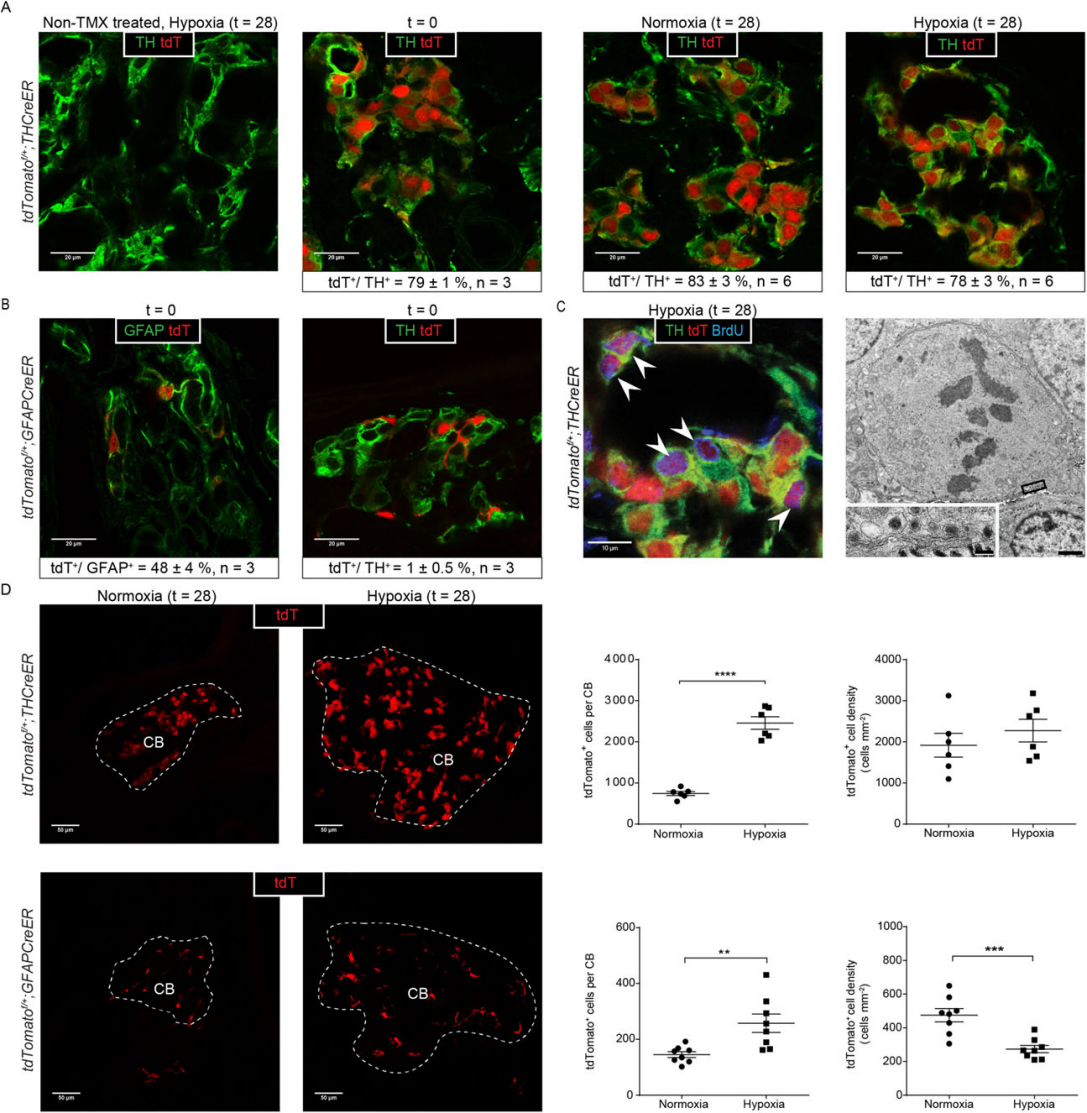
The effect of chronic hypoxia on lineage labelled Type I and Type II cells in the CB Representative images and morphometric analyses of lineage labelled *tdTomato^f/+^;THCreER* mice (A, C and D) and *tdTomato^f/+^;GFAPCreER* mice (B and D). A, characterization of tdTomato expression (tdT, red) and TH (green) immunostaining; far left: non‐tamoxifen (TMX) treated mice exposed to hypoxia (28 days at 10% oxygen); middle to far right: analysis immediately after tamoxifen ‘washout’ (*t* = 0), and normoxic or hypoxic mice after a further 28 days (*t* = 28); morphometric analyses on each are presented below. B, characterization of tdTomato (red) with GFAP (green) or TH (green) immunostaining immediately after tamoxifen ‘washout’ (*t* = 0); morphometric analyses on each are presented below. C, proliferation in hypoxic Type I cells; colocalization of BrdU^+^ immunostaining and tdTomato fluorescence (purple cells, marked with white arrowheads) in hypoxic *tdTomato^f/+^;THCreER* mice. Far right: electron microscopy image of a Type I cell (confirmed via the presence of DCVs; see insert) undergoing mitosis from a mouse exposed to 7 days of hypoxia. Scale bars = 1 μm (low power) and 100 nm (high power). D, representative images and quantification of tdTomato fluorescence in the CBs (CB edge outlined in white) of hypoxic mice (28 days at 10% oxygen) *vs*. normoxic controls (28 days normoxia) in *tdTomato^f/+^;THCreER* mice and *tdTomato^f/+^;GFAPCreER* mice. Values shown are the mean ± SEM. ^**^ *P* < 0.01, ^***^ *P* < 0.001, ^****^ *P* < 0.0001.

For each transgene, we aimed to activate the reporter gene by administration of tamoxifen for 5 days. This was followed by a 5 day ‘washout’ period to allow clearance of the drug, before animals were exposed to an atmosphere of 10% oxygen for 28 days. Thus the expansion, or otherwise, of the marked populations and their progeny could be analysed and compared with control animals maintained in normoxia. A number of control experiments were performed to ensure the integrity of the lineage marking strategy. First, we confirmed tamoxifen‐restricted activation of the lineage markers: tdTomato expression was not detectable in *tdTomato^f/+^;THCreER* non‐tamoxifen‐treated animals. Because the TH gene has been reported to be induced by hypoxia (Czyzyk‐Krzeska *et al*. 1992), we also examined the effect of the 28 day hypoxic exposure on *tdTomato^f/+^;THCreER* animals that had not received tamoxifen (Fig. 1 *A*, far left). Again, no fluorescent cells were detected, indicating that recombinant activation of the lineage markers was tightly restricted by tamoxifen under the conditions of our experiments. Second, we confirmed the anticipated lineage specificity of the lineage marker activation prior to hypoxic exposure. These experiments were performed in animals treated with tamoxifen for 5 days and then allowed to ‘washout’ for 5 days [i.e. at a time equivalent to the beginning (*t* = 0) of the hypoxic exposure]. This enabled exact comparison of any alteration in the morphology or associated immunoreactivity after 28 days of hypoxia with the position prior to that exposure (Fig. 1 *A*).

At *t* = 0, *tdTomato^f/+^;THCreER* animals manifest large numbers of tdTomato positive (tdTomato^+^) Type I cells, as defined by typical morphological appearances (i.e. large cells with rounded nuclei arranged in a glomerular architecture), confirming the anticipated location of the lineage marker (Fig. 1 *A*, middle left). To further confirm this and to estimate recombination efficiency, sections of CBs were immunostained with an anti‐TH antibody (Fig. 1 *A*, middle left). Confocal microscopy was used to assess colocalization of TH immunoreactivity and tdTomato: morphologically; by the use of Z‐stack planar analyses; and by the appearance of a perinuclear halo of yellow, derived from co‐incidence of nuclear tdTomato and cytoplasmic green fluorescence signals from anti‐TH (Fig. 1 *A*, middle left). Twenty images from each of three pairs of CBs were analysed, indicating that tdTomato fluorescence was essentially confined (>99%) to TH positive (TH^+^) Type I cells (Fig. 1 *A*, middle left). Conversely, ∼80% of TH^+^ cells were tdTomato^+^ (Fig. 1 *A*), indicating a high efficiency of recombination. Similarly, and as expected at *t* = 0, *tdTomato^f/+^;GFAPCreER* animals manifest tdTomato fluorescence in Type II cells, as defined by irregular morphology, small nuclear size and colocalization with anti‐GFAP immunoreactivity (Fig. 1 *B*, far left) (De Kock, 1951; Kameda, 2005). Fewer tdTomato^+^ cells were observed than in *THCreER* animals (Fig. 1 *A*, *B* and *D*), consistent with the previously described ratios of Type II *vs*. Type I cells in the CB (Kumar & Prabhakar, 2012). Confocal imaging, performed as above at *t* = 0, demonstrated that ∼50% of GFAP^+^ cells were tdTomato^+^, indicating a substantial, though lower, recombination frequency (Fig. 1 *B*, far left). These experiments therefore confirmed accurate lineage marking of CB cells, demonstrated that activation of the marker was tightly restricted to the 5 day exposure of animals to tamoxifen, and confirmed lineage restriction of the marker after tamoxifen ‘washout’ immediately prior to the 28 day experimental exposure to hypoxia.

To define the response of lineage marked Type I cells, *tdTomato^f/+^;THCreER* animals were exposed to 10% oxygen for 28 days or maintained in normoxia. This 28 day time point was selected as a time point that has been consistently demonstrated to result in significant hypoxia‐induced hyperplasia of the CB in rodent models (Laidler & Kay, 1975; Dhillon *et al*. 1984; McGregor *et al*. 1984). To identify proliferating cells, BrdU was administered via i.p. injection at *t* = 0 and added at 1 mg mL^−1^ to the drinking water for the duration of the experiment. As expected, hypoxia led to a substantial ∼3.5‐fold increase in CB size in hypoxic animals (0.0068 ± 0.0005 mm^3^ *vs*. 0.0019 ± 0.0004 mm^3^ in control animals, *P* < 0.0001). Analysis of these CBs revealed a commensurate 3‐fold increase in the number of cells expressing tdTomato fluorescence (2457 ± 152 *vs*. 745 ± 50 cells per CB, *P* < 0.0001) (Fig. 1 *D*, upper). Morphometric analyses of the CBs of hypoxic animals, using confocal microscopy and anti‐TH immunohistochemistry, revealed that essentially all (>99%) of this expanded number of tdTomato^+^ fluorescent cells manifested Type I cell morphology and/or co‐labelled with anti‐TH as had been observed in the *t* = 0 analyses, and as was also observed in CBs from control animals maintained in parallel in air (Fig. 1 *A*, right). Additional analyses indicated that, in each setting (*t* = 0, normoxia and hypoxia), the proportion of TH^+^ cells that were tdTomato^+^ was constant at ∼80% (Fig. 1 *A*). These observations suggest that there was little differentiation to or from the expanding Type I cell population in hypoxic animals. Further analysis using anti‐BrdU revealed BrdU incorporation in many, although not all, of the tdTomato^+^ cells (Fig. 1 *C*, left). Although quantitative assessment of the proportion of double (BrdU^+^/tdTomato^+^) labelled cells is difficult, definite co‐labelling was observed in the region of one‐third of the BrdU^+^ cells. Consistent with this, ultrastructural analyses identified dense core vesicle containing Type I cells undergoing mitosis (Fig. 1 *C*, right). In addition, we observed BrdU labelling in cells that were not tdTomato^+^ and vice versa. Taken together, these findings indicate that the Type I cell lineage expansion in hypoxia is substantially derived from the Type I cells that are present in the normoxic CB, and that this is driven by the proliferation of at least a proportion of such cells.

To define the response of lineage marked Type II cells, tamoxifen‐treated *tdTomato^f/+^;GFAPCreER* mice were similarly given BrdU and exposed to hypoxia for 28 days, or maintained in air after which CBs were examined using tdTomato fluorescence and immunostaining for BrdU and GFAP. These analyses revealed that this population also proliferated and expanded within the hypoxic CBs, but to a lesser extent than the Type I lineage marked population: 258 ± 33 *vs*. 145 ± 10 tdTomato^+^ cells per CB being observed in hypoxic animals *vs*. those maintained in air (Fig. 1 *D*, lower). In keeping with the smaller expansion in cell number *vs*. that of the Type I lineage marked population, the density of these cells appeared reduced in the enlarged hypoxic CBs (274 ± 22 *vs*. 475 ± 39 tdTomato^+^ cells mm^−2^, *P* = 0.0005) (Fig. 1 *D*, lower). Morphological examination and immunostaining with anti‐TH did not identify substantial transdifferentiation into Type I cells in either *t* = 0 or after 28 days of hypoxia or normoxia (Fig. 1 *B* and data not shown).

### Cell‐specific expression and inactivation of HIF‐α isoforms

**Figure 2:**
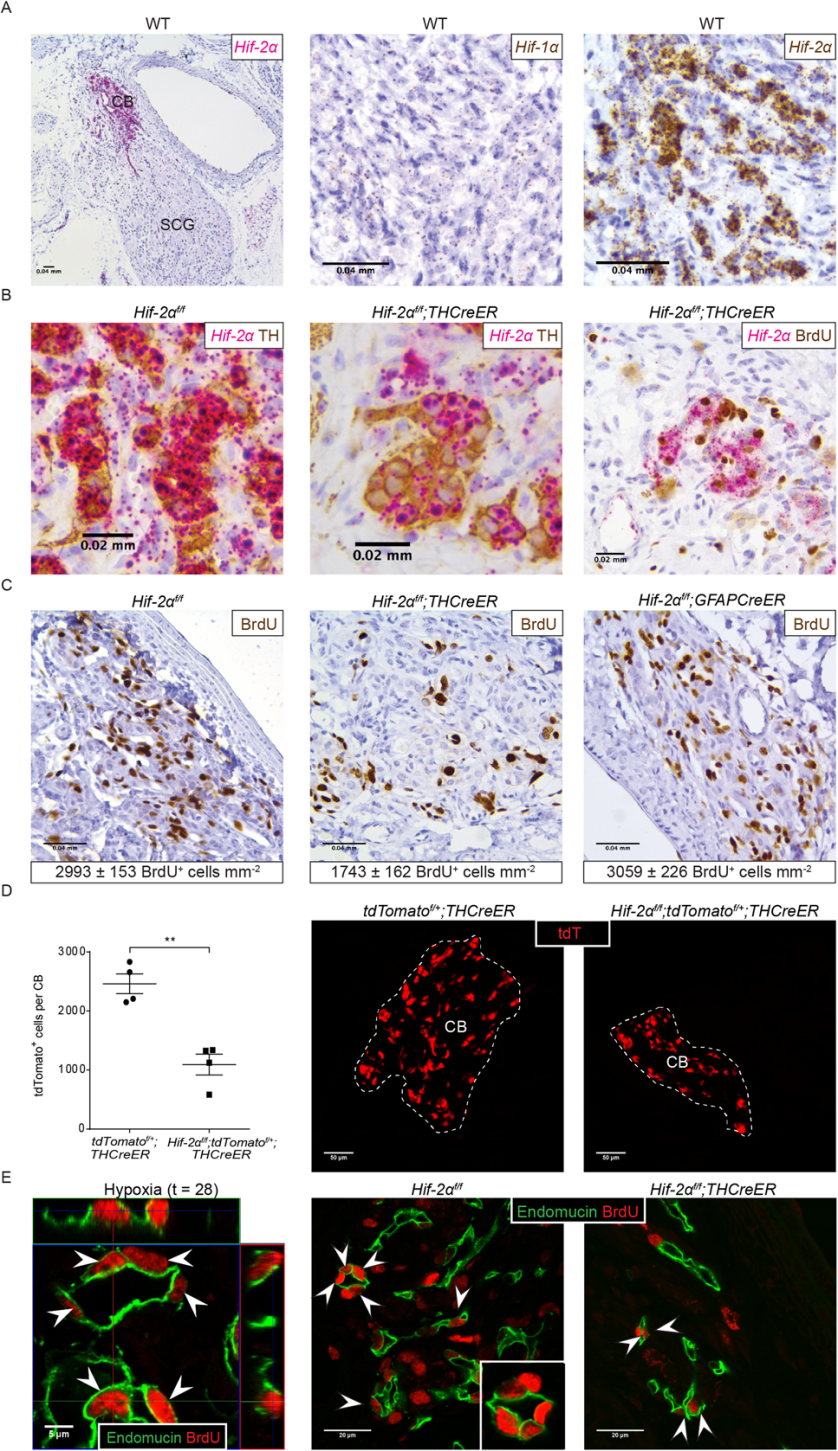
*Hif‐2α* mRNA expression in the CB and the effect of HIF‐2α inactivation on CB proliferation A, *in situ* hybridization for *Hif‐1α* and *Hif‐2α* mRNA in CBs from normoxic wild‐type (WT) mice. Low power image showing *Hif‐2α* expression (pink) around the carotid bifurcation, including the CB and superior cervical ganglion (SCG) (left). High power images of CB tissue showing *Hif‐1α* (middle) and *Hif‐2α* (right) expression (brown). B and C, representative images of CBs from *Hif‐2α^f/f^;THCreER*, *Hif‐2α^f/f^;GFAPCreER* and *Hif‐2α^f/f^* littermate control mice after exposure to 10% oxygen for 7 days. B, dual *in situ* hybridization and immunostaining for *Hif‐2α* mRNA (pink) and TH (brown) or BrdU (brown). C, immunostaining for BrdU (brown), morphometric analyses are presented below. Values shown are the mean ± SEM; *n* = 4–11. D, representative images and quantification of tdTomato^+^ cell numbers in the CBs (CB edge outlined in white) of hypoxic (28 days at 10% oxygen) *tdTomato^f/+^;THCreER vs. Hif‐2α^f/f^;tdTomato^f/+^;THCreER* mice; *n* = 4. Values shown are the mean ± SEM. ^**^ *P* < 0.01. E, endomucin (green) and BrdU (red) immunostaining (cells with colocalization are marked with white arrowheads) in mice. Left: *tdTomato^f/+^;THCreER* mice exposed to hypoxia (28 days at 10% oxygen) with orthogonal view of Z stacks showing colocalization; middle and right: images from *Hif‐2α^f/f^;THCreER* and *Hif‐2α^f/f^* littermate controls exposed to 7 days of chronic hypoxia.

The above findings demonstrate robust proliferation of Type I cells of the CB in response to hypoxic challenge. However, because hypoxia occurs across all cells of the organ, it was not possible to determine whether this is a cell‐autonomous response. In previous work, we demonstrated that general inactivation of HIF‐2α, but not HIF‐1α, in adult animals ablates the proliferation of cells within the CB that is otherwise observed during a 7 day exposure to 10% oxygen (Hodson *et al*. 2016). The importance of HIF‐2α, as demonstrated by these experiments, is consistent with recent reports of high levels of *Hif‐2α* mRNA in the whole CB (Zhou *et al*. 2016; Gao *et al*. 2017) and in isolated Type I cells (Zhou *et al*. 2016). To confirm these reports and extend them to a cellular level within the intact CB, we performed *in situ* hybridization for *Hif‐1α* and *Hif‐2α* mRNA within the CB and surrounding tissues. These experiments revealed a modest signal for *Hif‐1α* mRNA that was uniform across the tissues in contrast to a strong *Hif‐2α* mRNA signal that was tightly circumscribed within the CB (and mostly absent in neighbouring TH^+^ superior cervical ganglion and nerve fibres) and appeared to be particularly dense in clusters of cells morphologically typical of Type I cells (Fig. 2A). This was confirmed by dual detection of TH immunoreactivity (Fig. 2 *B*, left).

We therefore hypothesized that HIF‐2α activation by hypoxia within the CB Type I cells might drive the proliferation of that population. To test this hypothesis, we intercrossed *THCreER* with *Hif‐2α^f/f^* animals (*Hif‐2α^f/f^*;*THCreER*) and administered tamoxifen for 5 days to inactivate HIF‐2α in the Type I cells of CBs of adult animals. As found previously, a 5 day period of tamoxifen ‘washout’ was applied before mice were dosed with BrdU and exposed to sustained hypoxia (10% oxygen for 7 days) or maintained in air. Alterations in CB function were assessed at this time, using plethysmography to measure responsiveness to a short (5 min) hypoxic challenge, performed immediately after removal from sustained hypoxia; CB morphological changes were assessed by light and electron microscopy. The hypoxic responses of animals of each of the intercrossed genotypes were compared with those in controls that had been similarly exposed to tamoxifen but which only bore the *Hif‐2α^f/f^* alleles and therefore could not recombine. As before, BrdU incorporation was assessed using an anti‐BrdU antibody. The results are shown in Fig. 2 *C* and reveal a marked, but incomplete, reduction in BrdU incorporation (1743 ± 162 *vs*. 2993 ± 153 BrdU^+^ cells mm^−2^ in *Hif‐2α^f/f^;THCreER vs. Hif‐2α^f/f^*, *P* = 0.0006). Dual immunohistochemical labelling and *in situ* hybridization showed that cells expressing *Hif‐2α* mRNA, which colocalized with TH‐immunoreactivity, were much reduced (by ∼50%) in *Hif‐2α^f/f^*;*THCreER* animals, consistent with cell type specific inactivation of HIF‐2α (Fig. 2 *B*, middle). However, regions of *Hif‐2α* signal remained (Fig. 2 *B*, middle) and appeared to be associated with BrdU positive cells (Fig. 2 *B*, right), suggesting that the more moderate reduction in cell proliferation in these animals, *vs*. the previously reported effect of general inactivation of HIF‐2α (Hodson *et al*. 2016), is a result at least in part of incomplete recombination. To test whether this reduction in proliferation leads to a reduction in Type I cell number/CB size, we assessed the effects of 28 days of hypoxia on CBs from HIF‐2 α inactivated mice to facilitate comparisons with the lineage tracing experiments performed in Fig. 1 *D*. This shows a large reduction in labelled Type I cells (Fig. 2 *D*), as well as CB volume (0.0022 ± 0.0003 mm^3^ *vs*. 0.0068 ± 0.0003 mm^3^ in hypoxic *Hif‐2α^f/f^*;*tdTomato^f/+^;THCreER vs. tdTomato^f/+^;THCreER* controls, *P* < 0.0001), that significantly impairs the hypoxic expansion observed in Fig. 1 *D*. Overall, these findings suggest that the rapid proliferation and expansion of Type I cells in CBs of adult hypoxic mice is at least partially a cell‐autonomous/autocrine response that is dependent on HIF‐2α.

Our previous lineage tracing experiment indicated that many BrdU^+^ proliferating cells did not co‐label with tdTomato in either *THCreER* or *GFAPCreER* mice. These analyses revealed that many of these cells had appearances of endothelial cells and appeared to cluster around the CB vasculature. To confirm the endothelial origin of substantial numbers of these dividing cells, we used double immunohistochemical labelling of anti‐BrdU and anti‐endomucin on hypoxic mice. This demonstrated that ∼40% of the total BrdU^+^ cells in the proliferating CB co‐labelled with endomucin (Fig. 2 *E*, left, white arrowheads). Furthermore, tdTomato did not colocalize with endomucin in CBs from *tdTomato^f/+^;THCreER* mice, indicating that these proliferating endothelial cells were not derived from lineage labelled Type I cells. To assess whether the proliferation of this population of cells is also affected by the loss of HIF‐2α in Type I cells, we assessed the colocalization of BrdU and endomucin in CBs from hypoxic *Hif‐2α^f/f^;THCreER* mice and controls. The density of BrdU^+^/endomucin^+^ cells was significantly reduced in *Hif‐2α^f/f^;THCreER* mice relative to controls (762 ± 114 *vs*. 1256 ± 101 BrdU^+^/endomucin^+^ cells mm^−2^, *P* = 0.0174) (Fig. 2 *E*). This suggests that hypoxia‐induced endothelial cell proliferation is at least partially driven by HIF‐2α activation in the Type I cells, possibly via paracrine effects.

**Figure 3:**
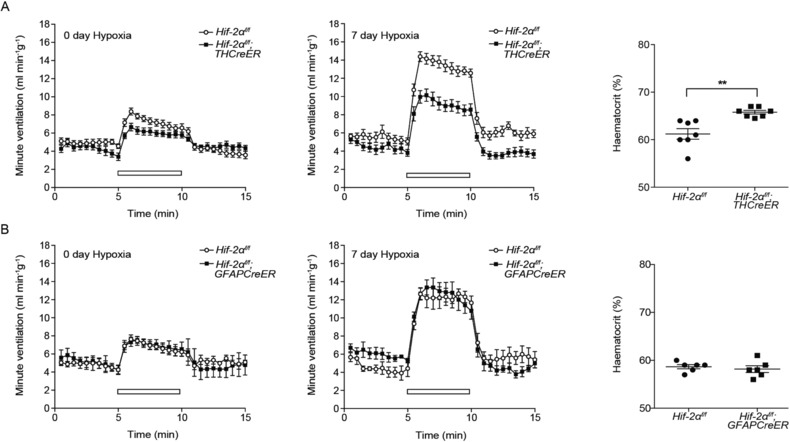
The effect of conditional HIF‐2α inactivation upon ventilatory responses and haematocrit levels in mice acclimatizing to chronic hypoxia A, minute ventilation before, during and after an acute hypoxic stimulus (10% oxygen with 3% carbon dioxide – white bars) and haematocrit measures of blood samples taken from *Hif‐2α^f/f^;THCreER* and *Hif‐2α^f/f^* littermate controls. Measurements made before (0 days of hypoxia) and after (7 days of hypoxia) acclimatization to 10% oxygen for 7 days. B, similar measurements from *Hif‐2α^f/f^;GFAPCreER* mice and littermate controls. Values shown are the mean ± SEM; *n* = 5–12. ^**^ *P* < 0.01.

Functional comparisons of the same animals (*Hif‐2α^f/f^*;*THCreER vs. Hif‐2α^f/f^*) revealed marked differences: the enhanced HVR, which was observed after 7 days of hypoxia in *Hif‐2α^f/f^* (control, unrecombined) animals, was much reduced in *Hif‐2α^f/f^*;*THCreER* animals (HVR of 9.13 ± 0.78 *vs*. 5.99 ± 0.42 mL min^−1^ g^−1^, *P* = 0.003) (Fig. 3 *A* and Table 1). This impaired ventilatory acclimatization to hypoxia was accompanied by a more severe polycythaemic response in *Hif‐2α^f/f^*;*THCreER^ ^*mice compared to *Hif‐2α^f/f^* controls (65.8 ± 0.4% *vs*. 61.2 ± 1.1% haematocrit, *P* = 0.002) (Fig. 3 *A*), consistent with an impact on systemic oxygen delivery.

**Table 1:** Acute hypoxic ventilatory responses of mice after conditional inactivation of HIF‐2α in TH or GFAP expressing cells before and after chronic hypoxia exposure

Acute hypoxic stimulus	Chronic hypoxia duration	Hypoxic ventilatory response (mL min^−1^ g^−1^)			ANOVA
		*Hif‐2α* ^*f/f*^	*Hif‐2α* ^*f/f*^;*THCreER*	*t* test *P* value	*P* value
10% O_2_	0 h	–0.05 ± 0.30	0.03 ± 0.34	0.858	
	48 h	2.86 ± 0.33	1.70 ± 0.34	**0.028**	**<0.0001**
	7 days	4.24 ± 0.21	2.84 ± 0.45	**0.013**	
ANOVA *P* value	**0.0069**				0.069
10% O_2_/3% CO_2_	0 h	3.32 ± 0.28	2.89 ± 0.30	0.314	
	48 h	6.43 ± 0.56	4.58 ± 0.46	**0.023**	**<0.0001**
	7 days	9.13 ± 0.78	5.99 ± 0.42	**0.003**	
ANOVA *P* value	**<0.0001**				0.012

Acute hypoxic stimulus	Chronic hypoxia duration	Hypoxic ventilatory response (mL min^−1^ g^−1^)			ANOVA
		*Hif‐2α* ^*f/f*^	*Hif‐2α* ^*f/f*^;*GFAPCreER*	*t* test *P* value	*P* value
10% O_2_	0 h	–0.24 ± 0.40	0.82 ± 0.57	0.167	
	48 h	1.30 ± 1.12	1.79 ± 0.85	0.804	**0.0004**
	7 days	3.70 ± 0.87	3.06 ± 0.42	0.530	
ANOVA *P* value	0.631				0.405
10% O_2_/3% CO_2_	0 h	3.15 ± 0.36	3.06 ± 0.31	0.844	
	48 h	6.66 ± 1.17	6.64 ± 0.49	0.987	**<0.0001**
	7 days	8.25 ± 1.02	7.51 ± 0.73	0.571	
ANOVA *P* value	0.647				0.873

Hypoxic ventilatory responses of *Hif‐2α^f/f^;THCreER* and *Hif‐2α^f/f^;GFAPCreER* mice *vs. Hif‐2α^f/f^* littermate controls. Measurements were taken after tamoxifen‐induced recombination and before (0 h) and after (48 h, 7 days) exposure to 10% oxygen. Values shown are mean ± SEM changes in minute ventilation in response to the indicated acute hypoxic stimulus; *n* = 5–12 littermate pairs for each genotypic comparison. Significance was tested using two‐way ANOVAs (right hand column *P* value = chronic hypoxia factor; bottom row *P* value = genotype factor; right column, bottom row *P* value = chronic hypoxia/genotype interaction factor), followed by *t* tests (with Holm–Sidak correction) for analysis of individual time points; *P* < 0.05 highlighted in bold.

Some reduction in HVR in *Hif‐2α^f/f^*;*THCreER^ ^*mice was detectable even prior to chronic hypoxic exposure, although this did not reach statistical significance (Fig. 3 *A* and Table 1). Reduced ventilatory responses under baseline conditions have been noted previously in other models of genetic inactivation of HIF‐2α: inducible, ubiquitous inactivation of HIF‐2α significantly reduced HVRs prior to chronic hypoxia and a similar, non‐significant trend was observed in *Hif‐2α^+/−^* mice (Hodson *et al*. 2016). Taken together, this suggests a role for HIF‐2α in the acute hypoxic ventilatory response, with the varying levels of residual HVR following inactivation of HIF‐2α perhaps reflecting the degree to which there is residual *Hif‐2α* mRNA/incomplete recombination.

Similar experiments performed on *Hif‐2α^f/f^*;*GFAPCreER vs. Hif‐2α^f/f^* animals revealed no differences in either the BrdU labelling, hypoxic ventilatory responses or haematocrit (Figs 2 *C* and 3 *B* and Table 1). Although these experiments do not support a major role for HIF‐2α within Type II cells in the overall response of the carotid to hypoxia, lower levels of *Hif‐2α* mRNA in these cells (Fig. 2 *B*) and a lower efficiency of recombination, as assessed by the tdTomato reporter gene experiments described above (Fig. 1 *B*), make it difficult to draw a firm negative conclusion.

**Figure 4:**
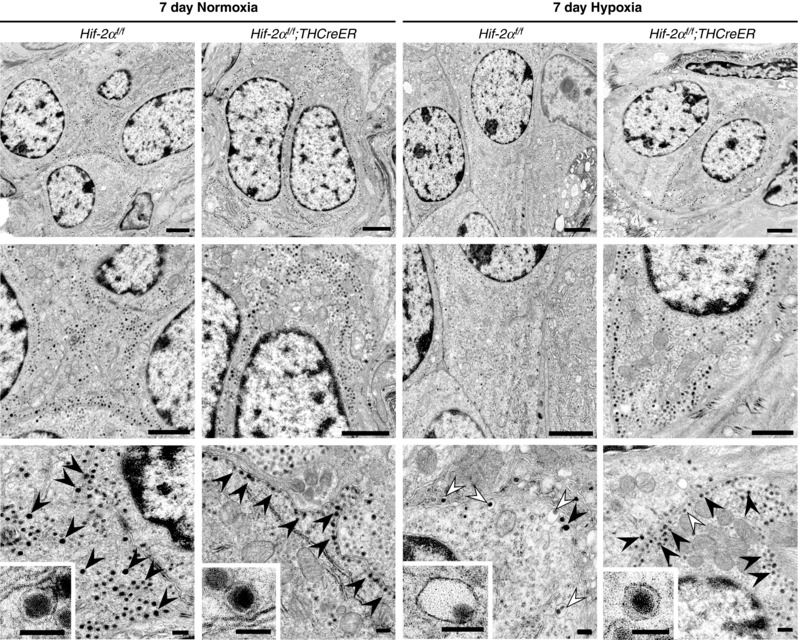
The effect of chronic hypoxia and HIF‐2α inactivation upon Type I cell ultrastructure Electron microscopy imaging of Type I cells from CBs of *Hif‐2α^f/f^*;*THCreER* mice and *Hif‐2α^f/f^* littermate controls exposed to 7 days of 10% oxygen (*n* = 7) or normoxia (*n* = 3). Upper: low power electron microscope images showing the general appearance of Type I cells. Middle: intermediate magnification showing the presence of large numbers of DCVs in the Type I cells of both normoxia samples (left) and in the hypoxic *Hif‐2α^f/f^*;*THCreER* mice (far right) in contrast to the few DCVs present in the hypoxic *Hif‐2α^f/f^* littermate control (middle right). Lower: high magnification showing typical DCVs (black arrowheads) and eccentric DCVs (marked with white arrowheads). Note the increased proportion of eccentric to normal DCVs in the hypoxic *Hif‐2α^f/f^* (middle right) but not hypoxic *Hif‐2α^f/f^*;*THCreER* mice (far right). Representative and eccentric DCVs shown in inserts. Scale bars = 1 μm (upper and middle) and 100 nm (lower and inserts).

However, given that both functional changes and proliferative responses to 7 days of chronic hypoxia within the Type I cells of the CB appeared to be dependent on the high levels of HIF‐2α in these cells, we next aimed to determine whether inactivation of HIF‐2α affected the integrity of Type I cells as well as their number. Accordingly, we compared the ultrastructural appearances of the Type I cells in *Hif‐2α^f/f^* and *Hif‐2α^f/f^*;*THCreER* animals after 7 days of hypoxia or maintenance in normoxia (Fig. 4). In animals maintained in normoxia, as anticipated, Type I cells contained large numbers of dense core vesicles (DCVs) aligned close to the plasma membrane (Fig. 4). In animals exposed to hypoxia, marked changes in these vesicles were observed, including a reduction in DCV density in hypoxic *vs*. normoxic *Hif‐2α^f/f^* controls (1.37 ± 0.14 *vs*. 7.20 ± 0.25 DCVs μm^−2^, *P* < 0.0001) and the appearance of large ‘eccentric’ DCVs with a lucent ‘halo’ around the dense core (Fig. 4, lower, insert). Strikingly, in many Type I cells from *Hif‐2α^f/f^;THCreER* mice, these hypoxia‐induced changes in dense core vesicles were almost completely absent (4.45 ± 0.94 *vs*. 1.37 ± 0.14 DCVs μm^−2^ in hypoxic *Hif‐2α^f/f^*;*THCreER vs. Hif‐2α^f/f^* mice, *P* = 0.0121) (Fig. 4). Consistent with incomplete recombination, the alterations in hypoxia‐induced ultrastructural changes were cell‐specific within the fields examined, with some cells retaining the usual hypoxia‐induced changes in dense core vesicle appearance (Fig. 4, right). These findings suggest that, within hypoxic Type I cells, HIF‐2α is required for both proliferation and for oxygen‐dependent vesicular functions, and that both changes might contribute to the enhanced hypoxic ventilatory responses observed after sustained hypoxia.

Further to these intracellular changes in dense core vesicles, Type I cells were noted on electron micrographs to undergo hypertrophy in response to hypoxia, with an increase in Type I cell size (mean cross sectional area 41 ± 4 *vs*. 64 ± 2 μm^2^ in normoxic *vs*. hypoxic *Hif‐2α^f/f^* mice, *P* < 0.01), as reported previously (Dhillon *et al*. 1984; Pequignot *et al*. 1984; Mills & Nurse, 1993). This hypertrophy was not observed following inactivation of HIF‐2α in Type I cells (64 ± 2 *vs*. 44 ± 4 μm^2^ in hypoxic *Hif‐2α^f/f^ vs. Hif‐2α^f/f^* ^;^ *THCreER* mice, *P* < 0.01).

### Inactivation of the HIF prolyl hydroxylase PHD2 in Type I cells of the CB

In animal cells, the levels of HIF‐α proteins, and hence the activity of HIF, is regulated by the oxygen‐dependent activity of the HIF prolyl hydroxylases: PHD1, PHD2 and PHD3 (Epstein *et al*. 2001; Berra *et al*. 2003; Appelhoff *et al*. 2004). Previous work from our laboratory identified PHD2 as the most important of these enzymes in regulating ventilatory sensitivity to hypoxia, CB volume and Type I cell number (Bishop *et al*. 2013; Hodson *et al*. 2016). These experiments used both constitutive hemizygous *Phd2^+/^* ^−^ animals (Bishop *et al*. 2013) and inducible general inactivation of PHD2 in adult animals (Hodson *et al*. 2016), although they did not address which cell type was responsible for the effects. Taken the findings reported above into consideration, this led us to investigate the possibility that inactivation of PHD2 within the Type I cells of the CB alone would be sufficient to drive enlargement of the CB. In these experiments, we used intercrosses between animals bearing a constitutively active, but Type I cell restricted, *THCre* recombinase transgene and animals bearing a homofloxed *Phd2* allele (*Phd2^f/f^;THCre*).

**Figure 5:**
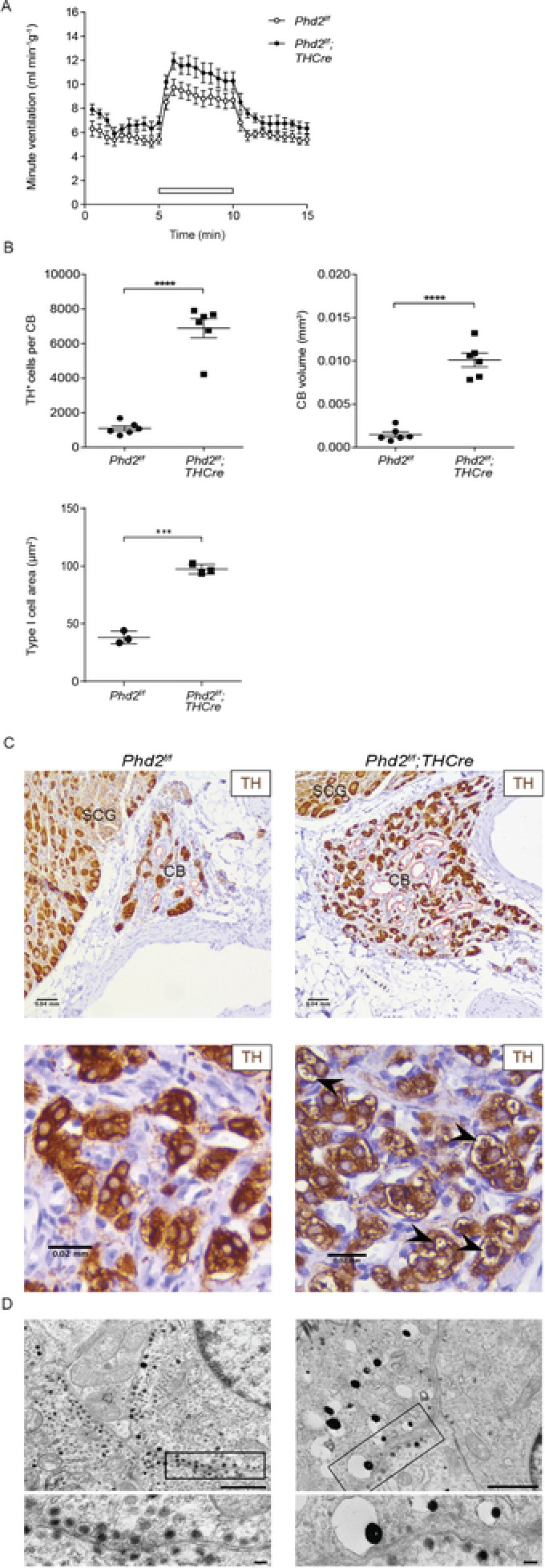
The effect of Type I cell‐specific PHD2 inactivation upon CB morphology and function A and B, CB morphology and ventilation in *n* = 6 *Phd2^f/f^;THCre* mice and *Phd2^f/f^* littermate controls. A, minute ventilation before, during and after an acute hypoxic stimulus (10% oxygen with 3% carbon dioxide – white bars). B, quantification of TH^+^ cell number and CB volume (from TH immunostaining in C); Type I cell area (from electron microscopy in D). C, representative images of TH (brown) immunostaining with vascularization marked in red. Low magnification images (upper) showing both the CB and the superior cervical ganglion (SCG). High magnification images (lower) showing the presence or absence of clearing in Type I cells (marked with black arrowheads). D, electron microscopy imaging of cellular ultrastructure and eccentric DCVs in Type I cells. Representative DCVs and eccentric DCVs shown in inserts. Scale bars = 1 μm (low power) and 100 nm (high power). Values shown are the mean ± SEM; ^***^ *P* < 0.001, ^****^ *P* < 0.0001.

When examined at 3 months of age, these *Phd2^f/f^;THCre* animals manifest slightly, but not significantly, increased baseline ventilation and hypoxic ventilatory responses compared to control (*Phd2^f/f^*) animals (Fig. 5 *A* and Table 2). By contrast, morphological changes in CBs were very striking (Fig. 5 *B* and *C*). The organs were greatly enlarged with multilineage hyperplasia involving large numbers of Type I cells embedded in organs that also manifested a large expansion of the vasculature (Fig. 5 *C*, upper**)**. Electron microscopy showed that Type I cells were surrounded by an expanded population of Type II cells in a glomerular arrangement; in addition to this hyperplasia, Type I (but not Type II) cells increased in cell size (Fig. 5 *B*) similar to, but greater than, that observed in hypoxia. Using light microscopy, a ‘clear cell’ phenotype was observed in approximately half of the hyperplastic CBs (Fig. 5 *C*, lower), similar to the vacuolated cytoplasm observed in carotid paragangliomas (Ashley & Evans, 1966; Robertson & Cooney, 1980; Wieneke & Smith, 2009) and enlarged CBs from high altitude dwellers (Edwards *et al*. 1971; Edwards *et al*. 1972; Arias‐Stella & Valcarcel, 1976). Cellular ‘clearing’ was manifest as large clear regions in Type I cells staining for neither lipid, nor glycogen. Electron microscopic analyses revealed that a large proportion of the Type I cells from *Phd2^f/f^;THCre* mice contained a relatively low density of dense core vesicles relative to controls (0.79 ± 0.09 *vs*. 8.82 ± 1.09 DCVs μm^−2^, *P* = 0.0018) but with increased vesicle diameter. In addition, a number of the Type I cells contained groups of larger eccentric vesicles (200–500 nm in diameter) with an eccentrically displaced dense core (Fig. 5 *D*). In these granules, the cores also appeared larger (150 nm *vs*. 90 nm in diameter) and more electron dense. Taken together, the reduced DCV number and appearance of eccentric DCVs was strongly reminiscent of the changes observed under electron microscopy in Type I cells exposed to chronic hypoxia (Fig. 4, middle right). The accumulation of large DCVs suggests that the clearing described above may represent the fusion of enlarged eccentric dense core vesicles, as reported previously in Type I cells obtained from guinea pigs at altitude (Edwards *et al*. 1972) and rats exposed to chronic hypoxia (Laidler & Kay, 1978).

**Table 2:** Acute hypoxic ventilatory responses in mice with inactivation of PHD2 in Type I cells combined with HIF‐1α or HIF‐2α inactivation

Genotype	Hypoxic ventilatory response (mL min^−1^ g^−1^)	
	10% O_2_	10% O_2_/3% CO_2_
*Phd2* ^*f/f*^	–0.31 ± 0.42	4.33 ± 0.62
*Phd2* ^*f/f*^;*THCre*	–0.41 ± 0.43	5.17 ± 0.51
*Phd2* ^*f/f*^;*Hif‐1α* ^*f/f*^;*THCre*	0.07 ± 0.68	4.07 ± 0.50
*Phd2* ^*f/f*^;*Hif‐2α* ^*f/f*^;*THCre*	–2.91 ± 0.50**§§	0.30 ± 0.62**§§
ANOVA *P* value	0.0017	0.0023

Mean ± SEM hypoxic ventilatory response values in age matched male mice measured at 3 months of age. Values shown are mean ± SEM changes in minute ventilation in response to the indicated acute hypoxic stimulus; *n* = 3–11 mice per genotype. Data were analysed by one‐way ANOVA followed by Tukey's multiple comparison test. ^**^ *P* < 0.01, significantly different from tamoxifen‐treated *Phd2^f/f^* controls; ^§§^ *P* < 0.01, significantly different from *Phd2^f/f^;THCre* mice.

**Figure 6:**
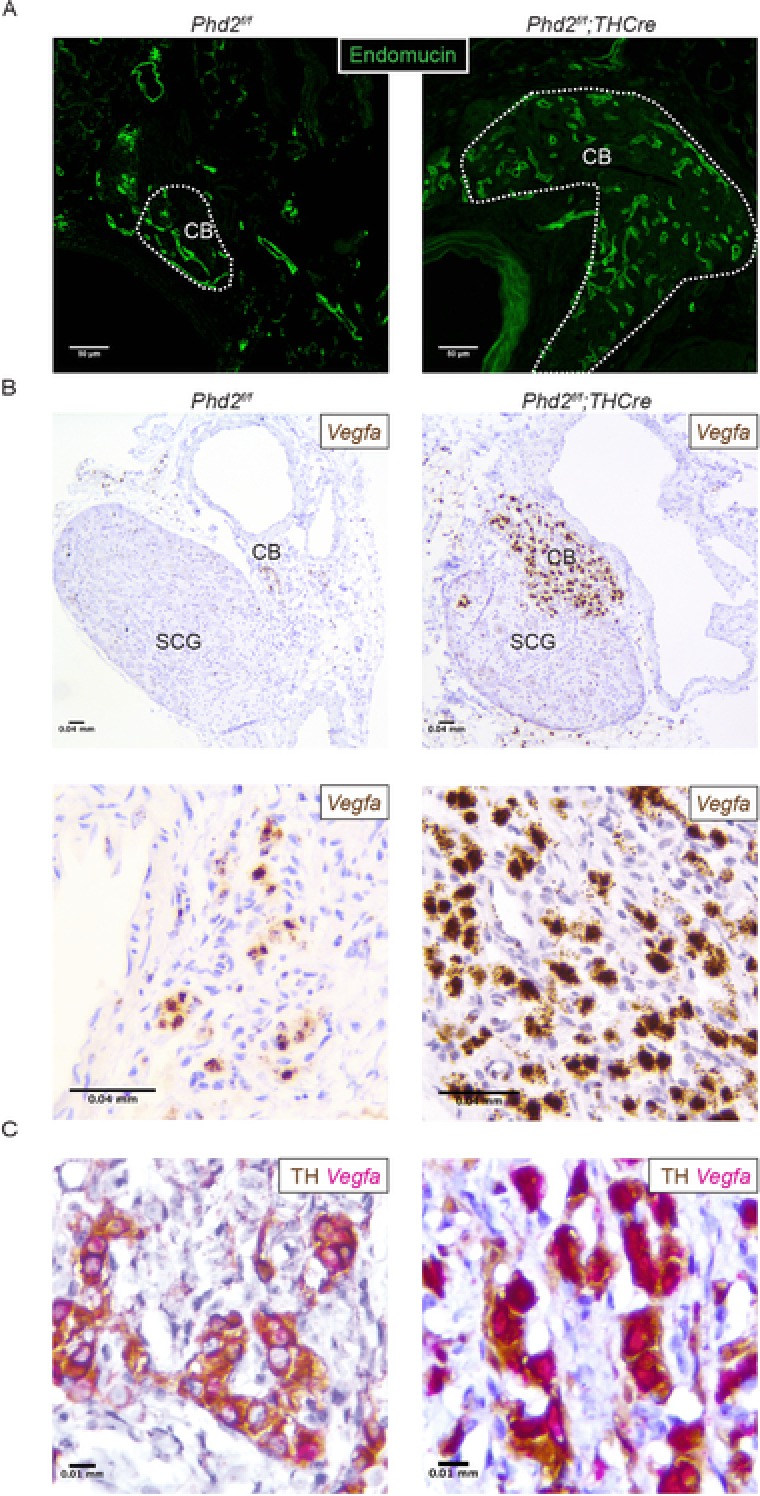
The effect of Type I cell‐specific PHD2 inactivation upon CB vasculature Representative images of CB tissues from *Phd2^f/f^;THCre* and *Phd2^f/f^* littermate controls. A, endomucin immunostaining (green, CB edges outlined in white). B, representative images of *Vegfa* mRNA (brown) *in situ* hybridization in the CB; low power images (upper) show both the CB and the superior cervical ganglion (SCG); high power images (lower) of the CB. C, *Vegfa* mRNA (pink) *in situ* hybridization combined with TH immunohistochemistry (brown).

These experiments indicate that inactivation of PHD2 within the Type I cells of the CB is sufficient to drive the generation of greatly enlarged CBs similar to, but greater than, the enlargement observed following chronic hypoxia (Fig. 5 *B vs*. Fig. 1 *D*). These morphological alterations clearly involved cells other than the Type I cells; in particular, the vasculature appeared to be greatly expanded (Fig. 5 *C*, upper). To confirm this, and to investigate potential mechanisms, we performed immunohistochemistry for the endothelial marker endomucin and *in situ* hybridization for vascular endothelial growth factor (*Vegfa*) mRNA (Fig. 6). These experiments confirmed the existence of a grossly hyperplastic endomucin^+^ vasculature (Fig. 6 *A*) and revealed a high level of expression of *Vegfa* mRNA, which was tightly confined to the CB and localized to Type I cells (Fig. 6 *B*), as identified by dual immunohistochemistry for TH (Fig. 6 *C*). Taken together, these findings suggest that activation of hypoxia signalling in Type I cells by reduced activity of PHD2 drives multilineage expansion within the CB by both cell‐autonomous or autocrine effects, as well as paracrine effects.

**Figure 7:**
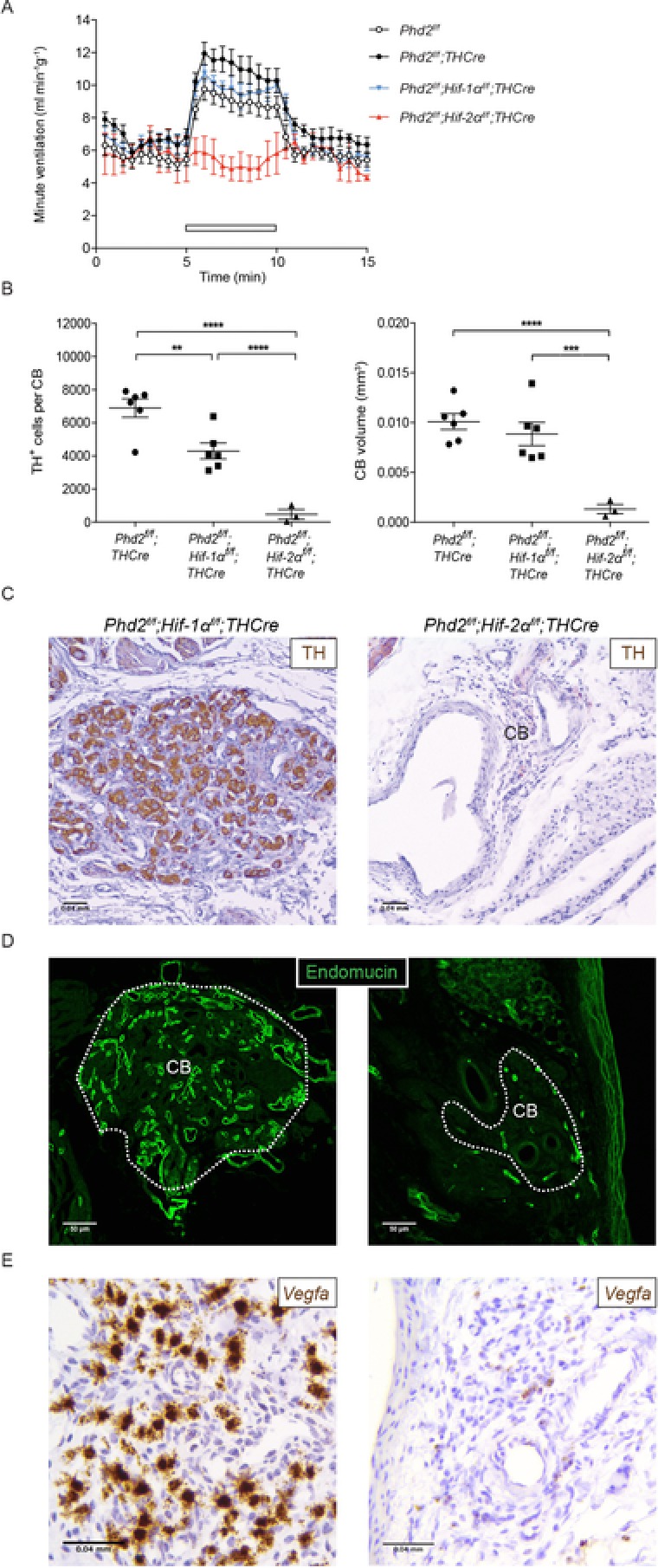
The effect of PHD2 inactivation in Type I cells combined with HIF‐1α or HIF‐2α inactivation upon CB morphology and function A, minute ventilation before, during and after an acute hypoxic stimulus (10% oxygen with 3% carbon dioxide – white bars) in *n* = 3–11 *Phd2^f/f^;THCre*, *Phd2^f/f^;Hif‐1α^f/f^;THCre*, *Phd2^f/f^;Hif‐2α^f/f^;THCre* mice and *Phd2^f/f^* littermate controls. B, quantification of TH^+^ cell number and CB volume from *Phd2^f/f^;THCre* (*n* = 6), *Phd2^f/f^;Hif‐1α^f/f^;THCre* (*n* = 6) and *Phd2^f/f^;Hif‐2α^f/f^;THCre* mice (*n* = 3). C–E, representative images of CBs from *Phd2^f/f^;Hif‐1α^f/f^;THCre* and *Phd2^f/f^;Hif‐2α^f/f^;THCre* mice. C, TH (brown) immunostaining. D, endomucin (green) immunostaining (CB edges in white). E, *in situ* hybridization for *Vegfa* mRNA (brown). Data from Fig. 5 *A* and *B* are reproduced for comparative purposes. Values shown are the mean ± SEM; ^**^ *P* < 0.01, ^***^ *P* < 0.001, ^****^ *P* < 0.0001.

Finally, given the importance of HIF‐2α in mediating CB responses to hypoxia in the adult, we aimed to determine whether these effects of long‐term PHD2 inactivation were mediated by either or both HIF‐α isoforms. Accordingly, *Phd2^f/f^;Hif‐1α^f/f^;THCre* and *Phd2^f/f^;Hif‐2α^f/f^;THCre* intercrosses were bred to generate animals with combined inactivation of PHD2 and HIF‐1α or HIF‐2α. As before, animals were examined at 3 months of age. The hypoxic ventilatory responses in animals with combined inactivation of PHD2 and HIF‐1α were similar to those observed with PHD2 inactivation alone (4.07 ± 0.50 *vs*. 5.17 ± 0.51 mL min^−1^ g^−1^) (Fig. 7 *A* and Table 2). By contrast, this HVR was markedly absent from *Phd2^f/f^;Hif‐2α^f/f^;THCre* mice (0.30 ± 0.62 *vs*. 5.17 ± 0.51 mL min^−1^ g^−1^, *P* < 0.01) (Fig. 7 *A* and Table 2) and these mice were intolerant of acute exposure to 10% oxygen (Table 2).

Moreover, these experiments revealed a minor decrease in hyperplasia when inactivation of PHD2 was combined with HIF‐1α, whereas some aspects of the phenotype, such as Type I cellular clearing and vascular expansion, appeared to be exacerbated (Fig. 7 *B*–*D*) or unchanged (e.g. hypertrophy of Type I cells: 97 ± 2 *vs*. 102 ± 3 μm^2^ in *Phd2^f/f^;THCre vs. Phd2^f/f^;Hif‐1α^f/f^;THCre* mice). By contrast, combined inactivation of PHD2 with HIF‐2α resulted in atrophic CBs in which Type I cell TH staining was almost absent and there was little or no vascular development (Fig. 7 *B*–*D*). In line with this, *in situ* hybridization for *Vegfa* showed very high *Vegfa* mRNA expression in CBs from *Phd2^f/f^;Hif‐1α^f/f^;THCre* mice and minimal *Vegfa* expression within the carotids of *Phd2^f/f^;Hif‐2α^f/f^;THCre* mice (Fig. 7 *E*). These results suggest that the phenotypes associated with pseudohypoxic PHD2 inactivation were dependent on HIF‐2α and are independent of, or exacerbated by, HIF‐1α inactivation.

## Discussion

The CB undergoes rapid cellular proliferation and hypertrophy in response to sustained hypoxia. This response is conserved across mammalian species and is associated with important functional changes, including enhanced ventilatory sensitivity to hypoxia (Edwards *et al*. 1971; Arias‐Stella & Valcarcel, 1973; Dhillon *et al*. 1984; McGregor *et al*. 1984; Heath *et al*. 1985; Bishop *et al*. 2013). However, despite intensive study, the mechanisms underlying these observations, including the interplay between functional changes and different proliferating cell populations, remain incompletely understood.

In the present study, we have used both lineage labelling technology and lineage‐restricted inactivation of specific components of the HIF prolyl hydroxylase system to investigate the cell type specific roles of that pathway within the CB. Our study reveals the importance of this system within the Type I cell lineage with respect to driving both proliferation and increased ventilatory sensitivity to hypoxia. First, using lineage marking technology, we demonstrate a large expansion of the Type I lineage in CBs studied after a period of 28 days sustained hypoxia. Second, we show that inactivation of the principal HIF prolyl hydroxylase PHD2, specifically within the Type I cells of the CB, is sufficient to drive multilineage hyperplasia and hypertrophy of the CB. Third, we show that the integrity of HIF‐2α, specifically within Type I cells, is required both for this response and for the proliferative changes that are observed upon exposure to sustained hypoxia. Taken together, these findings suggest that, within the CB, hypoxia acts on a population of Type I cells to drive rapid expansion of that lineage, and that the integrity of the PHD2–HIF‐2α couple is critical to that process.

A number of previous studies have reported on the origin of the cellular proliferation in hypoxic CBs. Although studies of the uptake of nucleoside analogues have reported labelling of Type I cells in hypoxic CBs (Wang & Bisgard, 2002; Wang *et al*. 2008), these studies do not directly trace the origin of such cells and labelling may reflect proliferation within another cell population that has then differentiated into Type I cells. In this context, evidence has been provided for the importance of glia‐like Type II cells, which act as a stem cell niche, and whose rapid amplification and differentiation could account for the expansion of multiple cell types, including Type I cells (Pardal *et al*. 2007). By directly tracing and characterizing the behaviour of lineage labelled Type I and Type II cells in response to sustained hypoxia, we demonstrate that the large majority of the Type I cell expansion that is observed in hypoxia derives from the Type I lineage itself and not via transdifferentiation from Type II cells. Although the Type II lineage labelled population of cells did expand in response to hypoxia, this expansion was smaller than that of the Type I cells. Although we cannot exclude a low level of transdifferentiation between Type I and Type II cells, or vice versa, we only observed occasional cells whose markers and morphology were compatible with this. Our results therefore appear to be at odds with the hypothesis that Type I cell expansion derives largely from a glia‐like stem cell niche (Pardal *et al*. 2007).

By contrast, when the manuscript of the present study was under preparation, a further report was published providing evidence for an immature Type I, TH‐expressing cell population that is capable of rapid division in response to hypoxia (Sobrino *et al*. 2018). These cells were distinguished by expression of the human natural killer 1 (HNK‐1) membrane epitope and manifest differences in their electrophysiological properties from mature Type I cells, although they contained secretory vesicles, were electrically excitable and expressed TH (Sobrino *et al*. 2018). Our findings are therefore consistent with this report and suggest that, in response to sustained hypoxia, this mode of Type I cell expansion (as opposed to transdifferentiation from glia‐like Type II cells) is quantitatively the more important route, at least under the conditions of our experiments.

Consistent with this, inactivation of PHD2 to create a ‘pseudohypoxic’ state specifically within Type I cells was sufficient to create hyperplasia of the CBs that resembles, but appears greater than (∼7‐fold *vs*. 3‐ to 4‐fold), that observed following chronic hypoxic exposure (Laidler & Kay, 1975; Dhillon *et al*. 1984; McGregor *et al*. 1984) or in long‐term dwellers at altitude (Edwards *et al*. 1971; Arias‐Stella & Valcarcel, 1973, 1976; Khan *et al*. 1988). By contrast to the significantly enhanced hypoxic ventilatory responses observed in mice following induced inactivation of PHD2 or 7 days of sustained hypoxia exposure (Bishop *et al*. 2013; Hodson *et al*. 2016), conditional inactivation of PHD2 in Type I cells did not result in significant changes in hypoxic ventilatory responses. Although this appears to be inconsistent with the greatly increased Type I cell number, it may reflect the long‐term effects of HIF‐2α stabilization, in a process that is possibly analogous to the hypoxic desensitization described in humans residing at high altitude (Severinghaus *et al*. 1966; Lahiri *et al*. 1976).

The enlarged CBs associated with Type I cell restricted PHD2 inactivation also manifest several features that are reminiscent of human paraganglioma (Ashley & Evans, 1966; Robertson & Cooney, 1980; Kliewer *et al*. 1989; Jyung *et al*. 2000; Van Den Berg *et al*., 2002; Wieneke & Smith, 2009). First, as well as expansion of the Type I population, they manifest marked expansion of endothelial cells and the vascular compartment. This indicates that signals originating from Type I cells were capable of affecting other lineages. Given that we did not observe transdifferentiation of Type I lineage marked cells to endothelial cells, the most probable possibility is that paracrine signals from Type I cells drive expansion of the CB vasculature. Although we have not formally demonstrated this, our observation of very high levels of *Vegfa* mRNA in Type I cells would be consistent with that hypothesis. Second, the enlarged CBs frequently showed a ‘clear cell’ phenotype by light microscopy, resembling the typical vacuolated histology of Type I cells in human paraganglioma (Ashley & Evans, 1966; Robertson & Cooney, 1980; Kliewer *et al*. 1989; Wieneke & Smith, 2009). Ultrastructural analysis revealed the presence of greatly enlarged vesicles with eccentrically displaced cores suggesting that these appearances arise, at least in part, as an extension of the enlargement of dense core vesicular structure that was evident after exposure to hypoxia. Thus, inactivation of PHD2 in a TH positive Type I cell population mimicked many of the effects of chronic exposure to hypoxia, with some abnormalities being more marked in the genetic condition perhaps because this ‘pseudohypoxic’ condition cannot be compensated for by increases in oxygen delivery. Interestingly, these results contrast with studies of VHL inactivation in Type I cells, which led to an almost total failure of CB development (Macias *et al*. 2014). It is possible that the actions of VHL and PHD2 inactivation are not equivalent because of non‐overlapping actions on systems other than HIF. However, an intriguing possibility is that the different outcomes arise from differences in quantitative effects on HIF activation (i.e. PHD2 inactivation causes a different and potentially lower level of HIF activation). Such a possibility would be consistent with human genotype–phenotype correlations in VHL disease where partial but not total loss of VHL function on the regulation of HIF is associated with development of pheochromocytoma (Kaelin, 2002, 2008).

It will require further studies to understand the precise means by which inactivation of PHD2 leads to the above phenotypes. The results of the present study in conjunction with previous work, however, support a key role for HIF‐2α within the Type I cell lineage. Activation of a stabilized form of HIF‐2α by expression of *THCre* has previously been shown to result in an expanded Type I cell population (Macias *et al*. 2014). In the present study, combined inactivation of HIF‐2α with PHD2 using the same *THCre* transgene entirely prevented CB overgrowth and led to a vestigial organ in which the Type I cells and associated vascular expansion were essentially absent. Another report published when the manuscript of the present study was under review demonstrated that inactivation of HIF‐2α under *THCre* also prevents CB development (Macias *et al*. 2018). Moreover, in the present study, we demonstrate that inducible inactivation of HIF‐2α in Type I cells in the adult was associated both with much reduced proliferation of Type I cells and with much reduced hypoxic ventilatory sensitivity.

These critical functions of HIF‐2α within the CB are consistent with the very high level of *Hif‐2α* mRNA within the organ demonstrated in both the present study and other studies (Tian *et al*. 1998; Zhou *et al*. 2016; Gao *et al*. 2017). Furthermore, our current *in situ* hybridization results are in accordance with single cell transcriptomic studies with respect to indicating tight restriction to the Type I cells (Zhou *et al*. 2016). By contrast, *Hif‐1α* mRNA is expressed at much lower levels. In keeping with this, the effects of combined HIF‐1α inactivation on the CB phenotype associated with PHD2 inactivation were more minor and not always in the same direction. Although some reduction in carotid overgrowth was observed in HIF‐1α/PHD2 *vs*. PHD2 conditionally inactivated mice, the ‘clear cell’ phenotype appeared to be exaggerated in HIF‐1α/PHD2 conditionally inactivated mice. Interestingly, somatic gain‐of‐function mutations in *HIF‐2α* but not *HIF‐1α* have been described in human paraganglioma (Toledo, 2017), including cases of Pacak–Zhuang syndrome, in which affected individuals manifest paraganglioma, as well as duodenal somatostatinoma and polycythaemia (Zhuang *et al*. 2012; Pacak *et al*. 2013). Thus, both mouse and human genetics point to a predominant role of HIF‐2α in CB hyperplasia.

Precisely how this relates to the organ's oxygen sensing function remains unclear. Interestingly, however, electron microscopy studies revealed that inactivation of HIF‐2α prevented hypoxia‐induced changes in both DCV number and appearance. This suggests that HIF‐2α is critical at some point on the pathway of hypoxia‐inducible vesicle release. Future studies aiming to investigate whether HIF‐2α regulates the acute oxygen sensitivity of Type I cells to trigger neurosecretion will be of interest.

Interestingly, it has been reported that blockade of calcium‐induced exocytosis impairs cell proliferation within the CB (Platero‐Luengo *et al*. 2014), raising the possibility of a link between the roles of HIF‐2α inactivation in Type I cell function and proliferation. At present, we cannot define precisely how increased proliferation relates back to the enhanced ventilatory responses to hypoxia, particularly because these effects were not concordant between the 7–28 day hypoxic exposures and long‐term PHD2 inactivation. Nor can we be certain that changes in ventilatory responses only reflect actions of HIF‐2α in the CB because the *THCre* mouse strains that were used have the potential to drive recombination in other catecholaminergic cells. Nevertheless, assays of recombination using the lineage tracer tdTomato indicated that recombination within the CNS was very limited, with little or no signal within the catecholaminergic neurons of the brainstem that have also been implicated in ventilatory control.

Overall, our findings suggest that HIF‐2α within the Type I cell lineage of the CB plays a critical role both in its oxygen sensing function and in its growth in response to sustained hypoxia. The generation of a HIF‐2α‐dependent paraganglioma‐like phenotype following inactivation of PHD2 provides a model for study of the pathogenesis of these tumours and may provide insights into the role of these pathways in promoting tumour growth in other settings.

## Additional information

### Competing interests

CWP and PJR are scientific co‐founders of ReOx Ltd, a university spin‐out company that aims to develop HIF hydroxylase inhibitors for therapeutic use.

### Author contributions

JWF, EJH, CWP, KJB, PJR and TB designed and conceived the experiments. JWF, EJH, XC, DJPF, LE, PL, MM, IR, CWP, KJB, PJR and TB collected, analysed and interpreted the data. JWF, EJH, PJR and TB drafted the article. JWF and EJH constructed the figures. JWF, EJH, XC, DJPF, LE, JA, PL, MM, IR, CWP, KJB, PJR and TB revised the manuscript critically for important intellectual content. All authors have read and approved the final copy submitted for publication. All experiments were conducted at the University of Oxford.

### Funding

The work was supported by the Wellcome Trust (grant number 106241/Z/14/Z) and the Ludwig Institute for Cancer Research. This work was also supported by the Francis Crick Institute which receives its core funding from Cancer Research UK (FC001501), the UK Medical Research Council (FC001501), and the Wellcome Trust (FC001501).

**Translational perspectives**

The present study implicates specific components of the HIF hypoxia signalling pathway in both rapid and long‐term responses of the carotid body to hypoxia. Using lineage tracing technology and cell type restricted recombination, the study reveals the cell‐autonomous operation of these molecules (PHD2/HIF‐2α) within the Type I lineage, thus opening the way to examine the targets of the HIF pathway operating within these cells. Apart from providing insights into the molecular physiology of the carotid body and ventilatory control, the results of the study may inform on mechanisms underlying cell proliferative responses to hypoxia that occur in disease, including cancers that express high levels of HIF‐2α. The development of agents that specifically target HIF‐2α (Courtney *et al*. 2018) may enable treatment of paraganglioma‐like tumours and our findings provide a model for testing this hypothesis. Notably, these agents have been associated with occasional episodes of reduced blood oxygen saturation in patients who received them for the treatment of kidney cancer, consistent with an action on the physiology of oxygen delivery to the blood.
